# The Chameleon Effect: Characterization Challenges Due to the Variability of Nanoparticles and Their Surfaces

**DOI:** 10.3389/fchem.2018.00145

**Published:** 2018-05-07

**Authors:** Donald R. Baer

**Affiliations:** Environmental Molecular Sciences Laboratory, Pacific Northwest National Laboratory, Richland, WA, United States

**Keywords:** nanoparticles, nano-objects, stability, characterization plan, surface analysis, reproducibility, provenance information, ISO Standard 20579-4

## Abstract

Nanoparticles in a variety of forms are increasing important in fundamental research, technological and medical applications, and environmental or toxicology studies. Physical and chemical drivers that lead to multiple types of particle instabilities complicate both the ability to produce, appropriately characterize, and consistently deliver well-defined particles, frequently leading to inconsistencies, and conflicts in the published literature. This perspective suggests that provenance information, beyond that often recorded or reported, and application of a set of core characterization methods, including a surface sensitive technique, consistently applied at critical times can serve as tools in the effort minimize reproducibility issues.

## Introduction

There is an increasing awareness of reproducibility issues in many areas of science including those associated with materials, biological, computational, and chemical research (Peng, 2011; Begley and Ioannidis, 2015; Baker, 2016; Buriak et al., 2016; Harris, 2017). Inherent characteristics of nanoparticles (NPs) make them particularly susceptible to reproducibility challenges associated with their production, characterization, and delivery. The inconsistencies and conflicts caused by these challenges have stimulated editorials and commentaries (Grainger and Castner, 2008; Nel et al., 2015), scientific news items (Candace, 2006; Cressey, 2010), and journal articles (Baer et al., 2008, 2013; Crist et al., 2013; Pettibone et al., 2013; Petersen et al., 2014). The multi- and cross-disciplinary nature of research and development associated with nano-objects makes it difficult for many research teams to be knowledgeable about all of the important issues and to have the range of tools needed to address them. This perspective focuses on two inter-related aspects of NP properties that complicate production and application of NPs with consistent behaviors and outlines ways to improve reproducibility and reliability.

As the size of particles decrease to the nano-size range, the surface to volume ratio increases along with the importance of the surface energy. This increased importance of surface energy, along with quantum effects at the smallest sizes, causes a variety of behaviors that make NP properties different from bulk forms of the same materials including: increased solubility as particle size decreases (Hochella, 2002), changes in stable crystal structure (Finnegan et al., 2007), enhanced adsorption of molecules from the environment (Jones et al., 2013), and particle growth or agglomeration (Wu et al., 2011). These processes and other phenomena impact particles in multiple ways including: (i) particle synthesis is often complex and not easily reproducible (Laban, 2017) and (ii) particles are unstable, easily damaged and frequently change as a function of time or environment (Baer et al., 2008, 2013; Karakoti et al., 2012). In earlier publications (Baer et al., 2013, 2016)we have identified these two inter-related issues as: (i) **NPs are not (usually) created equal** and (ii**) NPs are dynamic (like chameleons)**: they can change with time, handling, and environmental conditions. Sometimes very slight changes in a process or environmental condition cause particles to have unexpected behaviors after synthesis or at a later time. These behaviors are significant causes of reproducibility and inconsistency issues in studies involving NPs.

## Nanoparticle inequality

There are multiple major and minor ways that synthesis routes produce NPs with differing characteristics. Consequently any NP (single material particles or complex multi-layered particles) should be considered as a specific product produced by a specific process and not representative of all nanoparticles of that nominal type and size. As an easy example, Fe metal-core oxide-shell NPs were examined as a way to reduce environmental contaminants. Both reaction rates and pathways were found to vary with particles nominally of the same size but produced by different processes (Nurmi et al., 2005). It turns out that the particles produced by the two processes had significantly different internal and shell structures. We also found that Fe metal-core oxide-shell NPs produced by the same process, but using different starting salts, produced NPs with significantly varying reaction rates and pathways (Moore et al., 2011). A similar effect has been observed for ceria NPs. Altering the precursor salts during NP synthesis changed the biological outcomes (Barkam et al., 2017). For both these NPs, major or subtle differences in synthesis routes influenced NP properties.

**Figure 1:**
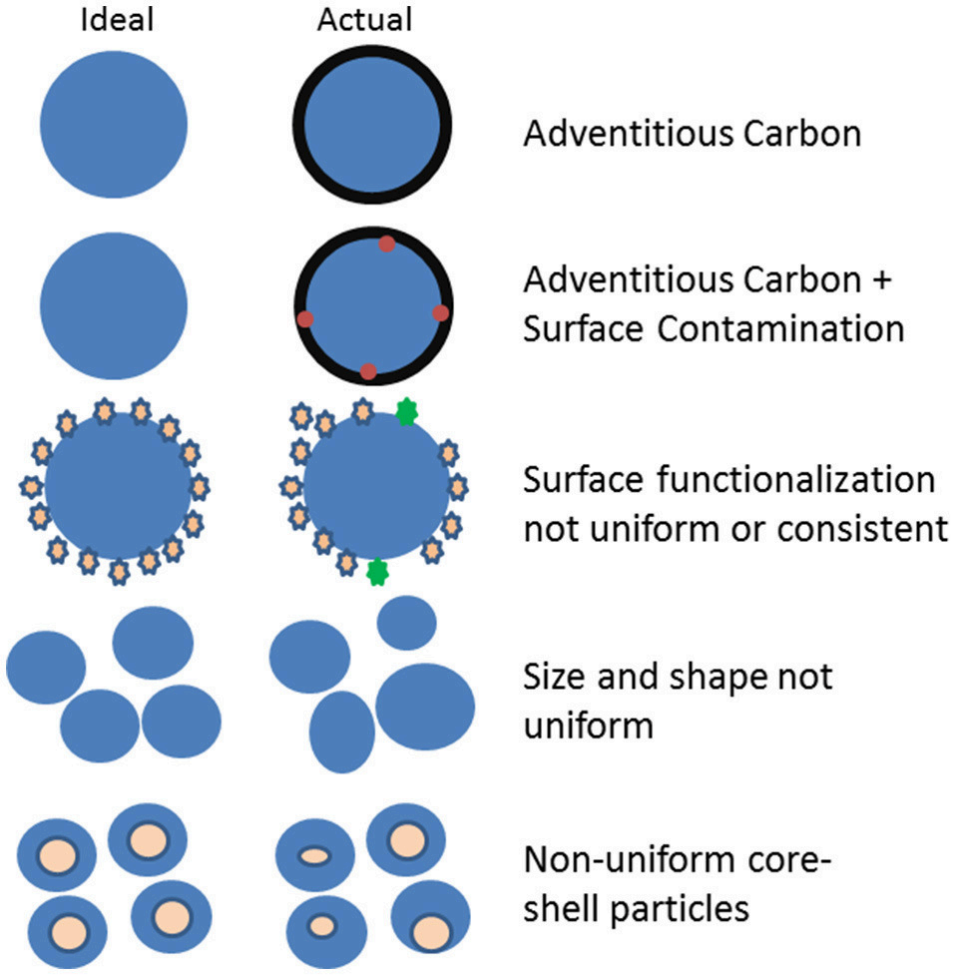
Examples of ways that NPs differ from the ideal or intended synthesis process.

As suggested in Figure 1, there are a variety of ways that particles as synthesized may differ from the “ideal” or intended synthesis result, all of which have been observed in our work and work of others. We have examined citrate stabilized Ag (Wang et al., 2016) and Au NPs (Techane et al., 2011) coated with a self-assembled monolayer (SAM) and in each case the presence of an adventitious carbon contamination layer was present along with the desired functional layer. Some of the Ag NPs had been formed around Au cores in an effort to produce NPs monodisperse in size. However, high angle annular dark-field transmission electron microscopy (HAADF-TEM) and X-ray photoelectrso spectroscopy (XPS) measurements provided information indicating that the Au cores were often not in the center of the particles and that the shapes and sizes were not as uniform as intended (Munusamy et al., 2015; Wang et al., 2016). Of more significance, Ag NPs with and without the Au core had different internal structures and dissolved at different rates in cell culture media (Munusamy et al., 2015).

Because of the high surface to volume ratio, surfaces have a significant impact on NP properties. It is unusual for NPs not to have some type of surface coating, adventitious, or deliberate. Surface coatings are often applied to stabilize particle dispersion and impart desired functionality. Studies done in the Sacher group have demonstrated the difficulty of reproducing, both commercially and in the laboratory, consistent functional coatings on superparamagnetic iron oxide NPs (SPIONs) (França et al., 2013; Mireles et al., 2016). Using XPS as an important surface sensitive analysis tool, the group found that for some types of functionalization it was nearly impossible to get consistent elemental and chemical state composition from batch to batch even when the coatings were added by the same person using the same chemicals, glassware, and procedure. Importantly, their work demonstrated the high importance of using surface analysis methods such as XPS to identify batch to batch variation of the particles surfaces. XPS measurements can identify unexpected elements (contamination) or chemical states variations or verify consistency of sample surfaces from batch to batch.

The presence of unplanned or unexpected elements on the surface is likely more common than generally reported by the research community. The Sacher group noted the presence of a wide variety of impurities on SPIONs made by different commercial processes (Mireles et al., 2016). As part of a study in the EMSL user facility examining Cu-oxide NPs to be used for toxicology tests, XPS identified the unexpected presence of F from the breakdown of PTFE during the synthesis process (Baer et al., 2013). Although XPS is increasingly used for analysis of NPs, many NP studies do not include any type of surface characterization by which unexpected surface contamination or other variations in particle surfaces might be identified.

These examples demonstrate some of the difficulties in producing NPs with reproducible properties. The description of a new product for producing NPs notes multiple challenges in NPs synthesis ending with “reproducibility is also a big issue” (Laban, 2017). Major, subtle and unintended variations in the synthesis process all serve to produce NPs whose properties may have unexpected variations. One team of researchers in our laboratory had produced ceria NPs for multiple years as graduate students. None-the-less, when these researchers tried to produce particles using the same processes in a different laboratory, particles with significantly less stability were produced (Karakoti et al., 2012).

As discussed in a later section, the collection and reporting of information that is often not recorded or reported can be one tool to help discover and minimize reproducibility issues. Such information can provide tracking records of sample history (provenance information) that helps identify possible sources of particle variations (Baer et al., 2016). Well-characterized NPs from a curated source such as the JRC Nanomaterials Repository (Cotogno et al., 2016) provides a way to obtain NPs with important provenance information.

## NPs are dynamic (like chameleons): they can change with time, handling, and environmental conditions

**Figure 2:**
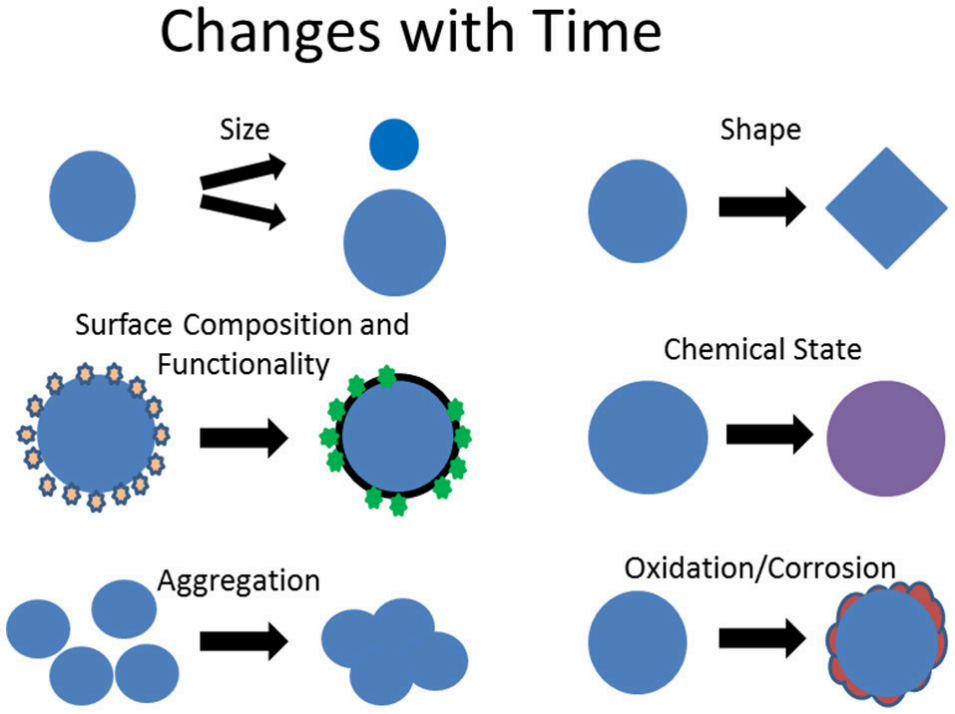
Examples of how NPs can change as a function of time or in different physical, chemical, or biological environmental conditions.

In discussing NPs for drug delivery systems, Wu et al. (2011) identify stability as a critical aspect in ensuring safety and efficacy of drug products using NPs. Impacts of time, processing, and environmental conditions on NPs—sometimes called aging—can take many forms (Baer et al., 2008; Mitrano et al., 2015) some of which are noted in Figure 2. NPs melt at lower temperatures than bulk material and can melt or change shape during many types of analysis (Smith et al., 1986; Yacaman et al., 2001; Zhao et al., 2006). Particles can become oxidized (Baer et al., 2008; Sarathy et al., 2008) and cycle between oxidation states (Kuchibhatla et al., 2012).

As indicated earlier, the high surface energy of NPs drives processes which lower the effective particle energy. Particles can be stabilized by the sorption of molecules from the surroundings or by lowering surface area by agglomeration or aggregation (Kraynov and Müller, 2011). NPs can form aggregates when stored as dry powders or in solution. For biological and toxicity studies the behavior of NPs in relevant biological media is critically important. In such media, aggregation, adsorption, and dissolution can all be important because each can influence the impact of NPs on a biological system. It was found that Fe oxide NPs formed agglomerates in cell culture media influencing dose-response profiles during *in vitro* studies (Sharma et al., 2014). The addition of fetal bovine serum (FBS) minimized the agglomeration and increased the reliability of the dose response curves (Sharma et al., 2014). The addition of FBS to the media minimized the particle changes and increased the accuracy and reproducibility of the study. However, the influence of the FBS was more complex for AgNPs. Although the addition of FBS to cell culture media stabilized the suspension of Ag NPs in solution—NPs did not aggregate and fall out of suspension—the FBS addition enhanced Ag dissolution. Thus the FBS minimized one type of particle change, but increased another (Munusamy et al., 2015).

The adsorption of proteins in biological media is particularly interesting as the surface layers have been identified as protein corona. These corona may be tens of nm thick (Lynch et al., 2007) and the nature of the corona, including composition, depends on the particle size (Zhang et al., 2011) and changes as a function of time transitioning from a “soft” corona to a “hard” corona (Vilanova et al., 2016). It is surface coatings on NPs that actually interact with the surrounding media, other particles, or a biological system (Jones et al., 2013). In almost all circumstances NPs turn into core-shell particles where the nature of the shell depends upon the environment.

In addition to changes in size and surface composition or structure due to dissolution, aggregation, corrosion, or sorption processes, we have observed changes in the chemical state of ceria NPs (Kuchibhatla et al., 2012). Ceria NPs are often grown in solution and the color of the solution can indicate the oxidation state of the particles. Particles identical in size, as examined by TEM after removal from solution, changed the solution color from yellow (presence of Ce^+4^) to clear (Ce^+3^) as a function of time. Nucleation of these particles had been initiated by addition of an oxidizer (H_2_O_2_) to a salt solution. As the oxidizing power of the solution lowered the particles switched from the +4 to the +3 state (Kuchibhatla et al., 2012). Changes or variations in the oxidation state of ceria NPs have also been observed within intact hydrated cells and organelles (Szymanski et al., 2015).

Although much of this perspective focuses on the preparation and delivery of well-defined NPs to minimize variability in NP studies, it is relevant to recognize that NPs can change at various times in the lifecycle of particles. This is particularly relevant to understanding behaviors in biological and environmental systems. Enhanced dissolution and transformations of Ag and ZnO NPs have been observed to occur at specific locations within cells (Liu et al., 2012; Mihai et al., 2015; Theodorou et al., 2017). Because of the time and effort involved, examination of transformations that occur within biological, environmental, and some materials systems would be accelerated by the development and advancement of the ability to make *in situ* real-time measurements (Sarathy et al., 2008; Szymanski et al., 2015).

Handling and cleaning of NPs prior to use or as preparation for analysis can also influence their surface composition and reactivity. In the case of AuNPs stabilized in citrate aqueous solutions, cleaning process such as centrifugation and resuspension or dialysis were observed to impact the surface functionalization efficiency (La Spina et al., 2017). The relative ease of particle transformation can be understood in terms of energetics [see Figure 6 of reference (Baer et al., 2008)]. NPs have been described as having protein like behaviors (Pelaz et al., 2012) in their size dependence and susceptibility to environmental influences, including preparation for analysis (La Spina et al., 2017). The lowered stability also makes NPs vulnerable to damage during analysis. Electron beams have been observed to influence particle shape (Yacaman et al., 2001), change crystal structure (Zhao et al., 2006), and induce oxidation (Wang et al., 2007).

The dynamic nature of NPs has several important implications: (i) the nature of nanoparticles cannot be assumed to be constant but must be examined or verified near the time of application; (ii) such behavior may impact storage times and lifetimes of products containing NPs, and (iii) susceptibility to damage has implications for both preparation of NPs for analysis and possible changes during analysis.

## Minimizing reproducibility issues

Challenges inherent in the production of consistent NPs along with the ease by which many types of NPs can change (the chameleon effect) provides an important backdrop to addressing NP reproducibility. The ability of seemingly insignificant changes in the synthesis process, handling, or other aspects of sample history to change NP properties highlights both (i) the need for a **high degree of care** in their **production and handling** along with **detailed records** about the pedigree of any batch of material and (ii) the need for **timely relevant characterization**. These two topics are discussed under the headings of **provenance information** and **characterization plan**.

### Provenance and provenance information

The concept of provenance is usually applied to authentication of the origin of a work of art. However, the concept can readily apply to other areas. The World Wide Web Consortium (W3C) established a Provenance Incubator Group that described the general concept as:

*Provenance of a resource is a record that describes entities and processes involved in producing and delivering or otherwise influencing that resource. Provenance provides a critical foundation for assessing authenticity, enabling trust, and allowing reproducibility (W3C*, *2010**)*.

With some minor adaptions to make it specific to NPs, the International Organization for Standards (ISO) description of *provenance information* is:

*Information that documents the history of a batch of NPs: This information tells the origin or source of the NPs, any changes that may have taken place since origination, and who has had custody of it since it was originated. Examples of provenance information are the principal investigator who recorded the data, and information concerning storage, handling, and migration* [adapted from ISO/TS 13527:2010, 1.4.2.36].

The clear objective of provenance information as applied to NPs is to provide a means to assess and validate properties and behaviors of a batch of material. The collection, retention, and reporting of provenance information can provide a tool to assist addressing NP reproducibility issues. As discussed previously (Baer et al., 2016) appropriate provenance information may vary with the particle type and application but likely includes:

- Record(s) of sample synthesis: reference or details of synthesis as known (e.g., process, vendor, lot number, chemicals, and their sources)
- Characterization results: data reports including relevant dates and processing of samples for analysis
- Important dates and times: synthesis, arrival in laboratory, opening of sample container, primary analysis measurements, and expiry date
- Storage time, conditions, and containers: temperature, humidity, media, light shielded, shipping, or transport.
- Record of additional processing: e.g., dried, washed, heated, sonicated, functionalized (including the method, dates, and number of times processed).

Much of this information is beyond that normally recorded or reported in the literature. We have started reporting times between synthesis, analysis, and application in our journal publications. ISO standard ISO TS 20579-4 has been developed indicating information to be added to a provenance information record regarding preparation of NPs for surface analysis. This standard also highlights the issues involved and discusses approaches to sample preparation for reliable surface analysis (ISO-20579-4, 2018).

### Characterization plan

The results of NP characterization are clearly an essential component of provenance information. Some of the long lists of desired measurements of NP properties can be very costly and, as discussed in the literature, might not improve material reproducibility (Hassellöv et al., 2008; Boverhof and David, 2010; Stefaniak et al., 2013). In our work on Fe metal-core oxide-shell nanoparticles we initially analyzed particles with a wide range of capabilities (Nurmi et al., 2005). Once we understood the behavior of the particles in many conditions we found that the routine application of three methods provided much of the needed information (Baer et al., 2013). These included measurement of particle size, shape, and structure using transmission electron microscopy and x-ray diffraction and surface composition using XPS.

The characterization needs and resulting plan for any set of NP materials will depend on the material, application and how well the material is understood (Baer et al., 2013). However, plan elements likely include:

- Establishment and routine application of a core set of characterization methods providing important information relevant to the specific application and materials (Baer et al., 2013).
  ◦ Methods used to prepare material for analysis (drying, dilution, filtering, cleaning etc.) should be applied in a consistent documented manner and recorded as part of the provenance information.
  ◦ Well-characterized particles, including those available in the JRC Nanomaterials Repository (Cotogno et al., 2016) are good starting points for materials with documented sample history and handling information.

- Some type of surface sensitive analysis tool needs to be applied to test for consistency and changes in NP surfaces (Baer et al., 2010).
  ◦ As suggested in Figures 1, 2, changes in surface composition, contamination, and functionalization are very common sources of particle variation.
  ◦ SESSA and other computational methods allow XPS to be used in a quantitative manner to extract important information about particle coatings and layer thicknesses (Powell et al., 2018).

- Stability and variability issues make measurement timing important. Some type of NP characterization or property validation measurement needs to be conducted close in time to the application or use of particles to verify consistency or minimal variation.
  ◦ As one example, we confirm particle size and zeta potential consistency immediately prior to *in vitro* studies.
  ◦ Determining the rates of particle change in relevant environments can identify appropriate storage times or handling conditions and potentially minimize unnecessary measurements and avoid surprises.
  ◦ For multiple reasons, including shelf time and batch to batch variation, it is unwise to assume that commercial materials have/retain the properties described or measured by a vendor (Crist et al., 2013).

Because of the importance of NP surfaces, as highlighted in the section Nanoparticle Inequality, including their impact on the interactions of particles (Jones et al., 2013) as well as the limited and frequently “sloppy” application of surface sensitive tools for NP characterization, it is particularly important to highlight the significant contributions that quality surface analysis can make to NP reproducibility (Grainger and Castner, 2008; Baer et al., 2013). The full range of surface analysis tools can be useful to understanding NP surfaces (Baer et al., 2010; ISO/TR-14187, 2011), but XPS has proven highly valuable for understanding the presence of contaminants and the uniformity of surface functionalization (Baer et al., 2013; Mireles et al., 2016), the chemical state of elements on NP surfaces including surface oxidation (Guascito et al., 2013), as well as the nature and thicknesses of surface and layered NP coatings (Techane et al., 2011; Shard, 2012; Belsey et al., 2015; Wang et al., 2016). Appropriate application of surface analysis methods requires careful and thoughtful sample preparation (ISO-20579-4, 2018), often involving extraction of samples for complex solution environments (Pourrahimi et al., 2014; La Spina et al., 2017) to be followed by appropriate data collection and spectral analysis (Guascito et al., 2013) that should include consideration of the potential impacts NP size and shape on the analysis (Baer and Engelhard, 2010; Powell et al., 2018).

## Conclusions/summary

The inherent nature of NPs causes challenges to their consistent production and application in reproducible studies. Awareness of the dynamical potential of many NPs (the chameleon effect) and the challenges of providing consistent surface chemistry can help research teams identify problems and find ways to improve consistency. The application of a well-defined and thought-out characterization plan, including some type of surface analysis, consistently applied at critical times, along with the collection and retention of provenance information (beyond that usually recorded or reported currently) can be useful tools to assist in addressing reproducibility issues or helping identify sources of possible variation.

Although there are significant sources of particle variation and needs for additional *in situ* tools to understand behaviors as they occur, with care and appropriate measurement feedback, much of the variability and inconsistencies in NP research can be significantly decreased.

## Author contributions

The author takes full responsibility for the views expressed in this perspective and has approved it for publication.

### Conflict of interest statement

The author declares that the research was conducted in the absence of any commercial or financial relationships that could be construed as a potential conflict of interest.

## References

[B1] Baer D. R. Amonette J. E. Engelhard M. H. Gaspar D. J. Karakoti A. S. Kuchibhatla S. (2008). Characterization challenges for nanomaterials. Surf. Interface Anal. 40, 529–537. 10.1002/sia.2726

[B2] Baer D. R. Engelhard M. H. (2010). XPS analysis of nanostructured materials and biological surfaces. J. Electron Spectros. Relat. Phenomena 178–179, 415–432. 10.1016/j.elspec.2009.09.003

[B3] Baer D. R. Engelhard M. H. Johnson G. E. Laskin J. Mueller K. Munusamy P.. (2013). Surface characterization of nanomaterials and nanoparticles: important needs and challenging opportunities. J. Vac. Sci. Technol. A 31:050820 10.1116/1.4818423 24482557 PMC3869349

[B4] Baer D. R. Gaspar D. J. Nachimuthu P. Techane S. D. Castner D. G. (2010). Application of surface chemical analysis tools for characterization of nanoparticles. Anal. Bioanal. Chem. 396, 983–1002. 10.1007/s00216-009-3360-1 20052578 PMC2841528

[B5] Baer D. R. Munusamy P. Thrall B. D. (2016). Provenance information as a tool for addressing engineered nanoparticle reproducibility challenges. Biointerphases 11:04B401. 10.1116/1.4964867 27936809 PMC5074995

[B6] Baker M. (2016). Is there a reproducibility crisis? Nature 533, 452–454. 10.1038/533452a 27225100

[B7] Barkam S. Ortiz J. Saraf S. Eliason N. Mccormack R. Das S.. (2017). Modulating the catalytic activity of cerium oxide nanoparticles with the anion of the precursor salt. J. Phys. Chem. C 121, 20039–20050. 10.1021/acs.jpcc.7b05725 28936278 PMC5602578

[B8] Begley C. G. Ioannidis J. P. A. (2015). Reproducibility in science: improving the standard for basic and preclinical research. Circ. Res. 116, 116–126. 10.1161/CIRCRESAHA.114.303819 25552691

[B9] Belsey N. A. Shard A. G. Minelli C. (2015). Analysis of protein coatings on gold nanoparticles by XPS and liquid-based particle sizing techniques. Biointerphases 10:019012. 10.1116/1.4913566 25724220

[B10] Boverhof D. R. David R. M. (2010). Nanomaterial characterization: considerations and needs for hazard assessment and safety evaluation. Anal. Bioanal. Chem. 396, 953–961. 10.1007/s00216-009-3103-3 19756533

[B11] Buriak J. M. Jones C. W. Kamat P. V. Schanze K. S. Schatz G. C. Scholes G. D. (2016). Virtual issue on best practices for reporting the properties of materials and devices. Chem. Mater. 28, 3525–3526. 10.1021/acs.chemmater.6b01854

[B12] Candace S. (2006). Particle size matters: studies fail to include basics for asserting toxicity. Small Times Magazine.

[B13] Cotogno G. Totaro S. Rasmussen K. Pianella F. Roncaglia M. Olsson H. (2016). The JRC Nanomaterials Repository - Safe Handling of Nanomaterials in the Sub-Sampling Facility. Ispra: Publications Office of the European Union.

[B14] Cressey D. (2010). Tiny traits cause big headaches: nanotech medicines held up by lack of particle characterization. Nature 467, 264–265. 10.1038/467264b 20844510

[B15] Crist R. M. Grossman J. H. Patri A. K. Stern S. T. Dobrovolskaia M. A. Adiseshaiah P. P.. (2013). Common pitfalls in nanotechnology: lessons learned from NCI's Nanotechnology Characterization Laboratory Integr. Biol. 5, 66–73. 10.1039/c2ib20117h 22772974 PMC3499664

[B16] Finnegan M. P. Zhang H. Banfield J. F. (2007). Phase stability and transformation in Titania nanoparticles in aqueous solutions dominated by surface energy. J. Phys. Chem. C 111, 1962–1968. 10.1021/jp063822c

[B17] França R. Zhang X. F. Veres T. Yahia L. H. Sacher E. (2013). Core–shell nanoparticles as prodrugs: possible cytotoxicological and biomedical impacts of batch-to-batch inconsistencies. J. Colloid Interface Sci. 389, 292–297. 10.1016/j.jcis.2012.08.065 23041026

[B18] Grainger D. W. Castner D. G. (2008). Nanobiomaterials and nanoanalysis: opportunities for improving the science to benefit biomedical technologies. Adv. Mater. 20, 867–877. 10.1002/adma.200701760

[B19] Guascito M. R. Chirizzi D. Malitesta C. Siciliano T. Tepore A. (2013). Te oxide nanowires as advanced materials for amperometric nonenzymatic hydrogen peroxide sensing. Talanta 115, 863–869. 10.1016/j.talanta.2013.06.032 24054675

[B20] Harris R. (2017). Reproducibility issues. Chem. Eng. News 95:2 Available online at: https://cen.acs.org/articles/95/i47/Reproducibility-issues.html

[B21] Hassellöv M. Readman J. W. Ranville J. F. Tiede K. (2008). Nanoparticle analysis and characterization methodologies in environmental risk assessment of engineered nanoparticles. Ecotoxicology 17, 344–361. 10.1007/s10646-008-0225-x 18483764

[B22] Hochella M. F. (2002). Nanoscience and technology the next revolution in the Earth sciences. Earth Planet. Sci. Lett. 203, 593–605. 10.1016/S0012-821X(02)00818-X

[B23] ISO/TR-14187 (2011). Surface Chemical Analysis – Characterization of Nanostructured Materials. International Organization for Standardization.

[B24] ISO-20579-4 (2018). Surface Chemical Analysis - Guidelines to Sample Handling, Preparation and Mounting - Part 4 - Reporting Information Related to the History, Handling and Mounting of Nano-Objects Prior to Surface Analysis. International Organization for Standadization.

[B25] Jones C. F. Castner D. G. Grainger D. W. (2013). Surface adsorbates on nanomaterials and their possible roles in host inflammatory and toxicological processing, in Handbook of Immunological Properties of Engineered Nanomaterials, eds Dobrovolskaia M. A. Mcneil S. E. (Hackensack, NJ: World Scientific Publishing Co. Inc), 117–149.

[B26] Karakoti A. S. Munusamy P. Hostetler K. Kodali V. Kuchibhatla S. Orr G. (2012). Preparation and characterization challenges to understanding environmental and biological impacts of ceria nanoparticles. Surf. Interface Anal. 44, 882–889. 10.1002/sia.5006 23430137 PMC3575181

[B27] Kraynov A. Müller T. E. (2011). Concepts for the stabilization of metal nanoparticles in ionic liquids, in Applications of Ionic Liquids in Science and Technology, ed Handy S. (London: InTech), 235–260. Available online at: https://www.intechopen.com/books/applications-of-ionic-liquids-in-science-and-technology/concepts-for-the-stabilization-of-metal-nanoparticles-in-ionic-liquids

[B28] Kuchibhatla S. V. N. T. Karakoti A. S. Baer D. R. Samudrala S. Engelhard M. H. Amonette J. E.. (2012). Influence of aging and environment on nanoparticle chemistry: implication to confinement effects in nanoceria. J. Phys. Chem. C 116, 14108–14114. 10.1021/jp300725s 23573300 PMC3618908

[B29] Laban V. (2017). Generating New Size-Specific Nanoparticles Within a Day [Online]. Available online at: https://www.azonano.com/article.aspx?ArticleID=4707 (Accessed January 8, 2018).

[B30] La Spina R. Spampinato V. Gilliland D. Ojea-Jimenez I. Ceccone G. (2017). Influence of different cleaning processes on the surface chemistry of gold nanoparticles. Biointerphases 12:031003. 10.1116/1.4994286 28750541

[B31] Liu J. Wang Z. Liu F. D. Kane A. B. Hurt R. H. (2012). Chemical transformations of nanosilver in biological environments. ACS Nano 6, 9887–99. 10.1021/nn303449n 23046098 PMC3508364

[B32] Lynch I. Cedervall T. Lundqvist M. Cabaleiro-Lago C. Linse S. Dawson K. A. (2007). The nanoparticle–protein complex as a biological entity; a complex fluids and surface science challenge for the 21st century. Adv. Colloid Interface Sci. 134–135, 167–174. 10.1016/j.cis.2007.04.021 17574200

[B33] Mihai C. Chrisler W. B. Xie Y. Szymanski C. Tolic A. Hu D.. (2015). Intracellular accumulation dynamics and fate of zinc ions in alveolar epithelial cells exposed to airborne ZnO nanoparticles at the air-liquid interface. Nanotoxicology 9, 9–22. 10.3109/17435390.2013.859319 24289294 PMC4652791

[B34] Mireles L.-K. Sacher E. Yahia L. H. Laurent S. Stanicki D. (2016). A comparative physicochemical, morphological and magnetic study of silane-functionalized superparamagnetic iron oxide nanoparticles prepared by alkaline coprecipitation. Int. J. Biochem. Cell Biol. 75, 203–211. 10.1016/j.biocel.2015.12.002 26667269

[B35] Mitrano D. M. Motellier S. Clavaguera S. Nowack B. (2015). Review of nanomaterial aging and transformations through the life cycle of nano-enhanced products. Environ. Int. 77, 132–147. 10.1016/j.envint.2015.01.013 25705000

[B36] Moore K. Forsberg B. Baer D. R. Arnold W. A. Penn R. L. (2011). Zero-valent iron: impact of anions present during synthesis on subsequent nanoparticle reactivity. J. Environ. Eng. Asce 137, 889–896. 10.1061/(ASCE)EE.1943-7870.0000407

[B37] Munusamy P. Wang C. Engelhard M. H. Baer D. R. Smith J. N. Liu C.. (2015). Comparison of 20 nm silver nanoparticles synthesized with and without a gold core: structure, dissolution in cell culture media, and biological impact on macrophages. Biointerphases 10:031003. 10.1116/1.4926547 26178265 PMC4506304

[B38] Nel A. E. Parak W. J. Chan W. C. W. Xia T. Hersam M. C. Brinker J. C.. (2015). Where are we heading in nanotechnology environmental health and safety and materials characterization? ACS Nano 9, 5627–5630. 10.1021/acsnano.5b03496 26100220

[B39] Nurmi J. T. Tratnyek P. G. Sarathy V. Baer D. R. Amonette J. E. Pecher K.. (2005). Characterization and properties of metallic iron nanoparticles: spectroscopy, electrochemistry, and kinetics. Environ. Sci. Technol. 39, 1221–1230. 10.1021/es049190u 15787360

[B40] Pelaz B. Jaber S. De Aberasturi D. J. Wulf V. Aida T. De La Fuente J. M.. (2012). The state of nanoparticle-based nanoscience and biotechnology: progress, promises, and challenges. ACS Nano 6, 8468–8483. 10.1021/nn303929a 23016700

[B41] Peng R. D. (2011). Peproducible research in computational science. Science 334, 1226–1227. 10.1126/science.1213847 22144613 PMC3383002

[B42] Petersen E. J. Henry T. B. Zhao J. Maccuspie R. I. Kirschling T. L. Dobrovolskaia M. A.. (2014). Identification and avoidance of potential artifacts and misinterpretations in nanomaterial ecotoxicity measurements Environ. Sci. Technol. 48, 4226–4246. 10.1021/es4052999 24617739 PMC3993845

[B43] Pettibone J. M. Gigault J. Hackley V. A. (2013). Discriminating the states of matter in metallic nanoparticle transformations: what are we missing? ACS Nano 7, 2491–2499. 10.1021/nn3058517 23425128

[B44] Pourrahimi A. M. Liu D. Pallon L. K. H. Andersson R. L. Martinez Abad A. Lagaron J. M. (2014). Water-based synthesis and cleaning methods for high purity ZnO nanoparticles - comparing acetate, chloride, sulphate and nitrate zinc salt precursors. RSC Adv. 4, 35568–35577. 10.1039/C4RA06651K

[B45] Powell C. J. Werner W. S. M. Kalbe H. Shard A. G. Castner D. G. (2018). Comparisons of analytical approaches for determining shell thicknesses of core-shell nanoparticles by x-ray photoelectron spectroscopy. J. Phys. Chem. C 122, 4073–4082. 10.1021/acs.jpcc.7b12070 PMC5990282 29887938

[B46] Sarathy V. Tratnyek P. G. Nurmi J. T. Baer D. R. Amonette J. E. Chun C. L. (2008). Aging of iron nanoparticles in aqueous solution: effects on structure and reactivity. J. Phys. Chem. C 112, 2286–2293. 10.1021/jp0777418

[B47] Shard A. G. (2012). A straightforward method for interpreting XPS data from core–shell nanoparticles. J. Phys. Chem. C 116, 16806–16813. 10.1021/jp305267d

[B48] Sharma G. Kodali V. Gaffrey M. Wang W. Minard K. R. Karin N. J.. (2014). Iron oxide nanoparticle agglomeration influences dose rates and modulates oxidative stress-mediated dose-response profiles *in vitro*. Nanotoxicology 8, 663–675. 10.3109/17435390.2013.822115 23837572 PMC5587777

[B49] Smith D. J. Petfordlong A. K. Wallenberg L. R. Bovin J. O. (1986). Dynamic atomic-level rearrangements in small gold particles. Science 233, 872–875. 17752214 10.1126/science.233.4766.872

[B50] Stefaniak A. B. Hackley V. A. Roebben G. Ehara K. Hankin S. Postek M. T.. (2013). Nanoscale reference materials for environmental, health and safety measurements: needs, gaps and opportunities. Nanotoxicology 7, 1325–1337. 10.3109/17435390.2012.739664 23061887

[B51] Szymanski C. J. Munusamy P. Mihai C. Xie Y. Hu D. Gilles M. K.. (2015). Shifts in oxidation states of cerium oxide nanoparticles detected inside intact hydrated cells and organelles. Biomaterials. 62, 147–154. 10.1016/j.biomaterials.2015.05.042 26056725 PMC4470772

[B52] Techane S. D. Baer D. R. Castner D. G. (2011). Simulation and modeling of self-assembled monolayers of carboxylic acid thiols on flat and nanoparticle gold surfaces Anal. Chem. 83, 6704–6712. 10.1021/ac201175a 21744862 PMC3165144

[B53] Theodorou I. Müller K. Chen S. Goode A. Yufit V. P. Porter A. (2017). Silver nanowire particle reactivity with human monocyte-derived macrophage cells: intracellular availability of silver governs their cytotoxicity. ACS Biomater. Sci. Eng. 3, 2336–2347. 10.1021/acsbiomaterials.7b00479 33445292

[B54] Vilanova O. Mittag J. J. Kelly P. M. Milani S. Dawson K. A. Rädler J. O.. (2016). Understanding the kinetics of protein–nanoparticle corona formation. ACS Nano 10, 10842–10850. 10.1021/acsnano.6b04858 28024351 PMC5391497

[B55] Wang C. M. Baer D. R. Amonette J. E. Engelhard M. H. Antony J. J. Qiang Y. (2007). Electron beam-induced thickening of the protective oxide layer around Fe nanoparticles. Ultramicroscopy 108, 43–51. 10.1016/j.ultramic.2007.03.002 17448600

[B56] Wang Y.-C. Engelhard M. H. Baer D. R. Castner D. G. (2016). Quantifying the impact of nanoparticle coatings and nonuniformities on xps analysis: gold/silver core–shell nanoparticles. Anal. Chem. 88, 3917–3925. 10.1021/acs.analchem.6b00100 26950247 PMC4821750

[B57] Wu L. Zhang J. Watanabe W. (2011). Physical and chemical stability of drug nanoparticles. Adv. Drug Deliv. Rev. 63, 456–469. 10.1016/j.addr.2011.02.001 21315781

[B58] W3C (2010). What is Provenance [Online]. Provenance Information Group. Available online at: https://www.w3.org/2005/Incubator/prov/wiki/What_Is_Provenance (Accessed 2016).

[B59] Yacaman M. J. Ascencio J. A. Liu H. B. Gardea-Torresdey J. (2001). Structure shape and stability of nanometric sized particles. J. Vac. Sci. Technol. B 19, 1091–1103. 10.1116/1.1387089

[B60] Zhang H. Burnum K. E. Luna M. L. Petritis B. O. Kim J.-S. Qian W.-J.. (2011). Quantitative proteomics analysis of adsorbed plasma proteins classifies nanoparticles with different surface properties and size. Proteomics 11, 4569–4577. 10.1002/pmic.201100037 21956884 PMC3252235

[B61] Zhao J. P. Chen Z. Y. Cai X. J. Rabalais J. W. (2006). Annealing effect on the surface plasmon resonance absorption of a Ti-SiO2 nanoparticle composite. J. Vac. Sci. Technol. B 24, 1104–1108. 10.1116/1.2188410

